# Supplementary material for: Identification of photoperiod-induced specific miRNAs in the adrenal glands of Sunite sheep (Ovis aries)
Source: Front Vet Sci. 2022 Jul 22;9:888207. doi: 10.3389/fvets.2022.888207 (PMC9354845; doi:10.3389/fvets.2022.888207)
Supplement: Supplementary file 1 [file Data_Sheet_1.ZIP › Supplementary file/miRDeep2 file.zip!/miRDeep2 file/Know_Structure/SPLP42A1/oar-mir-3955.pdf]

miRBase precursor : oar-mir-3955
Total read count : 507
oar-miR-3955-5p read count : 445
oar-miR-3955-3p read count : 46
remaining reads : 16

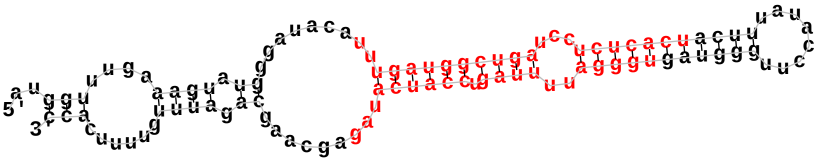

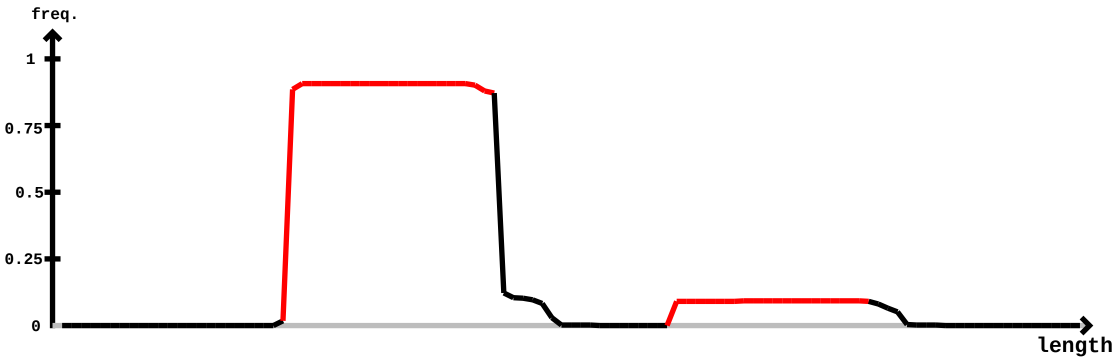

oar-miR-3955-5p

oar-miR-3955-3p

```
5'- augguuugaaaguaugggauacauuugauggcugauccucucacuacuuuauaccuuggguagugggauuuuaguccaucauagagcaagcagauuuguuuucacc   -3'    exp
    ...........((((...))))...((((((((((...(((((((((((........)))))))))))..)))).)))))).(((((((......)))))))....   reads   mm   sample
    ......................auuugauggcugauccucuca...............................................................   1       0    seq
    ......................auuugauggcugauccucucac..............................................................   1       0    seq
    ......................auuugauggcugauccucucacu.............................................................   6       0    seq
    ......................auuugauggcugauccucucacuacuu.........................................................   1       0    seq
    .......................uuugauggcugauccucuc................................................................   3       0    seq
    .......................uuugauggcugauccucuca...............................................................   9       0    seq
    .......................uuugauggcugUuccucuca...............................................................   1       1    seq
    .......................uuugauggcugauccucucac..............................................................   3       0    seq
    .......................uuugauggcugauccucucacu.............................................................   365     0    seq
    .......................uuugauggcugauccucucacua............................................................   8       0    seq
    .......................uuugauggcugauccucucacuac...........................................................   1       0    seq
    .......................uuugauggcugauccucucacuacu..........................................................   3       0    seq
    .......................uuugauggcugauccucucacuacuu.........................................................   6       0    seq
    .......................uuugauggcugauccucucacuacuuu........................................................   26      0    seq
    .......................uuugauggcugauccucucacuacuuua.......................................................   14      0    seq
    .......................uuugauggcugauccucucacuacuuuauacc...................................................   1       0    seq
    ........................ugauggcugauccucucacu..............................................................   9       0    seq
    ........................ugauggcugauccucucacua.............................................................   1       0    seq
    ........................ugauggcugauccucucacuacuuu.........................................................   1       0    seq
    ...............................................................ugggauuuuaguccaucaua.......................   1       0    seq
    ...............................................................ugggauuuuaguccaucauag......................   5       0    seq
    ...............................................................ugggauuuuaguccaucauaga.....................   8       0    seq
    ...............................................................ugggauuuuaguccaucauagag....................   7       0    seq
    ...............................................................ugggauuuuaguccaucauagagc...................   24      0    seq
    ...............................................................ugggauuuuaguccaucauagagca..................   1       0    seq
    ......................................................................uuaguccaucauagagcaagc...............   1       0    seq
```
